# Impact of Two Ant Species on Egg Parasitoids Released as Part of a Biological Control Program

**DOI:** 10.1673/031.013.10601

**Published:** 2013-10-07

**Authors:** Alan Kergunteuil, César Basso, Bernard Pintureau

**Affiliations:** 1 BF2I-UMR INRA/INSA de Lyon, INSA bâtiment L. Pasteur, 69621-Villeurbanne-cedex, France; 2 Facultad de Agronomía, Unidad de Entomología, av. Garzón 780, 12900-Montevideo, Uruguay

**Keywords:** Lepidoptera pests, *Pheidole bergi*, *Solenopsis richteri*, soybean, *Trichogramma*

## Abstract

Biological control using *Trichogramma pretiosum* Riley (Hymenoptera: Trichogrammatidae), an egg parasitoid wasp, was tested in Uruguay to reduce populations of lepidopteran pests on soybeans. It was observed that the commercial parasitoid dispensers, which were made of cardboard, were vulnerable to small predators that succeeded in entering and emptying the containers of all the eggs parasitized by *T. pretiosum*. Observations in a soybean crop showed that the only small, common predators present were two ant species. The species responsible for the above mentioned predation was determined from the results of a laboratory experiment in which the behavior of the two common ants was tested. A modification of the dispensers to prevent introduction of this ant has been proposed and successfully tested in the laboratory and in the field.

## Resumen

El control biológico para reducir las poblaciones de plagas de lepidópteros en soja utilizando *Trichogramma pretiosum* Riley (Hymenoptera: Trichogrammatidae), una avispa parasitoide de huevos, ha sido evaluado en Uruguay. Se observó que los difusores comerciales de parasitoides confeccionados en cartón eran vulnerables a pequeños depredadores que lograban entrar y vaciarlos de todos los huevos parasitados por este parasitoide. Observaciones en un cultivo de soja mostraron que los únicos depredadores pequeños presentes eran dos especies de hormigas. La especie responsable de la depredación anteriormente mencionada fue determinada a partir de los resultados de un experimento de laboratorio en el que se evaluó el comportamiento de las dos hormigas. Una modificación de los difusores para evitar la introducción en ellos de esta hormiga se propuso y evaluó con éxito en el laboratorio y en el campo.

## Introduction

Augmentative biological pest control is commercially applied worldwide by releasing large numbers of natural enemies over large crop areas (Lenteren 2000; Lenteren and Bueno 2003). *Trichogramma* species (Hymenoptera: Trichogrammatidae) are the most widely used parasitoids in inundative biological control of lepidopteran pests and are released by various means from the ground or air (Smith 1994). After their release, *Trichogramma* can remain in the host, inside the dispensers, for several hours or up to several days, during which time they are exposed to predators such as *Chrysoperla* *carnea* (Stephen) (Neuroptera: Chrysopidae) (Al Rouechdi and Voegelé 1981) or carnivorous ants. Over the years, a number of different techniques have been developed to protect or enclose the pre-emergent parasitoids, but problems persist.

**Figure 1.**
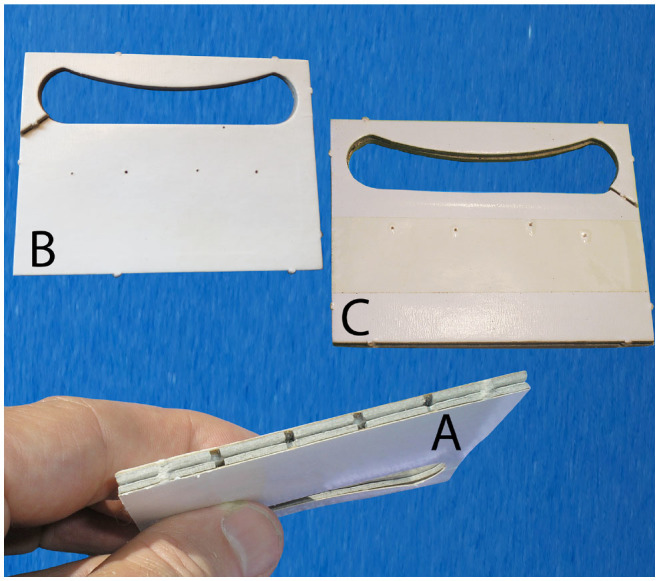
Dispensers used to release *Trichogramma pretiosum* in the field. A: usual dispenser with small holes on the edge; B: modified dispenser with very small holes on the side; C: modified dispenser improved with sticky tape at the level of the very small holes. High quality figures are available online.

In Uruguay, the release of *Trichogramma* *pretiosum* Riley is used to control the soybean pests *Rachiplusia mu* (Guénée) (Lepidoptera: Noctuidae) and *Anticarsia gemmatalis* (Hübner), according to an agreement between the Faculty of Agronomy (University of the Republic, Uruguay) and the agricultural businesses Barraca, J.W. Erro, and Tafilar, and with the help of the French company Biotop (Valbonne, France), which specializes in the production of parasitoids. During trials in January 2010, it was found that *T. pretiosum* suffered significant prédation in the dispensers (release systems for the parasitized host's eggs that are placed in fields, Figure 1A). The dispensers (6 × 8 cm), constructed of a waterproof cardboard envelope, contain eggs of *Ephestia kuehniella* Zeller (Lepidoptera: Pyralidae) parasitized by *T. pretiosum* at the pupal stage. After distribution of these dispensers on cultivated plants, *T. pretiosum* escape through the openings on the edges of the envelope.

Our study aimed to determine which predator species are responsible for predation of the parasitoid and to devise a simple solution to the problem. First, potential predators were collected and identified. Because of findings from previous studies, special attention was given to ant species (Marquier et al. 2009; Mgocheki and Addison 2009). An experimental device allowed the behavior of these organisms in the dispensers to be studied. Finally, whether a modification to the dispensers would be sufficient to protect *T. pretiosum* was tested. The laboratory results were verified in the field.

This problem of predation is especially important because soybean production is widespread in Uruguay (850,000 hectares (MGAP 2011)), and the control of soybean pests currently requires repeated application of excessive amounts of chemical insecticides. The use of biological control would reduce environmental pollution and better protect human health.

## Materials and Methods

### Collection of potential predators

As a result of inspections carried out during 24 February and 3 March 2010 in an experimental soybean field located in southwestern Uruguay, near Dolores city (33° 33′ S, 58° 12′ W), only two very common species of predators (ants) (Hymenoptera: Formicidae) were observed. In order to study the potential of these ants as *E. kuehniella's* egg predators, a single colony containing workers and brood of each species was dug up from the soil near plants containing predated dispensers and transported to the laboratory.

### Study of the behavior of potential predators in the laboratory

**Figure 2.**
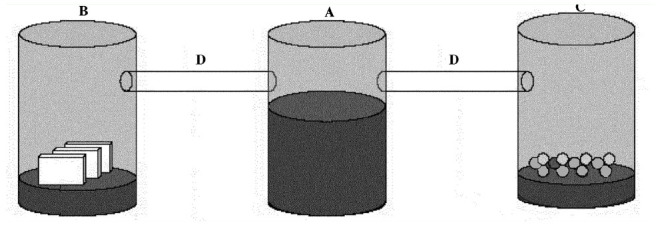
Experimental device made of transparent Perspex for the first experiment in the laboratory to study the behavior of potential predators of parasitized eggs. A: central compartment housing ants; B: compartment containing three *Trichogramma pretiosum* dispensers; C: compartment containing a mixture of seeds; D: tubes allowing the movement from one compartment to the other. The lower part of each compartment contained soil. High quality figures are available online.

Upon arrival at the laboratory, colonies containing about 1,000 workers were maintained at 23° C until the experiments were carried out (Castiñeiras 1981). Then, each colony was placed in an artificial nest consisting of three 2 L compartments aligned and interconnected by transparent tubes 1 cm in diameter (Figure 2).

The ants were kept in the central compartment for 15 hr before being connected to the other compartments. In order to investigate whether the two ant species prefer the food commonly found in nature or the added biological resource commonly used for biological control in crops, both food sources were available to the ants inside the two side compartments. One compartment contained three *T. pretiosum* dispensers containing *E*. *kuehniella* eggs (eggs were glued inside the dispensers using gum Arabic), and the other one contained a mixture of seeds not usually included in the dispensers (canary grass, *Phalaris canariensis* L. (Poales: Poaceae); oat, *Avena sativa* L., foxtail millet, *Setaria* *italica* L.; corn, *Zea mays* L.; *Panicum* spp.; flax, *Linum* spp.; sunflower, *Helianthus* *annuus* L. (Asterales: Asteraceae); rape, *Brassica napus* L. (Brassicales: Brassicaceae); canola, *Brassica rapa* L. The mixture contained an approximately equal number of each of the species that are more or less common in Uruguay for a total of 100 seeds in the compartment. No other seeds were added during the experiment. The quantity of food was not limiting. All experiments were conducted in a room maintained at 23° C.

The flow of ants in each tube (number of individuals/min) was measured toward dispensers and toward seeds for 1 minute every ½ hr or every 1 hr during a period of 8.5 hr. Two successive replicates were performed with the same ant colonies. Host egg density (number/mm^2^) in the dispensers was checked before exposure to ants, then 1 hour and several hours (3, 5, 7, 9, 24, 48, and 72 hr) after the start of ant exposure. These measurements were performed within 34 squares (each 32 mm^2^ and defined by a grid) selected at random over the entire surface covered by the eggs in each of the three dispensers (about 2,400 mm^2^ × 3). Data from the three dispensers were pooled.

### Laboratory test of modified dispensers to protect *Trichogramma pretiosum*

**Figure 3.**
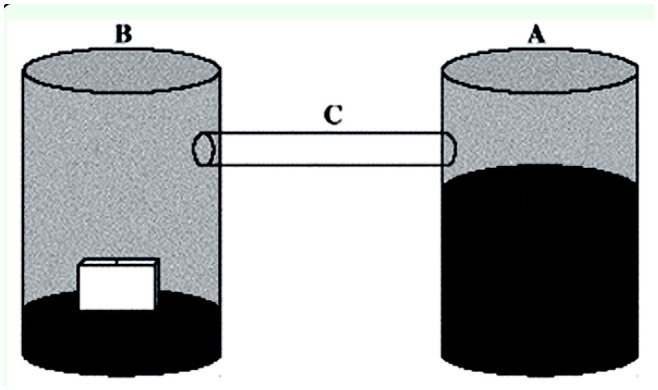
Experimental device made of transparent Perspex for the second experiment in the laboratory to test *Trichogramma pretiosum* protection in modified dispensers. A: compartment housing ants (only one species is studied); B: compartment containing two *T. pretiosum* dispensers, either the usual type or the modified type; C: tube allowing the movement between compartments. The lower part of the compartment contained soil. High quality figures are available online.

The device for testing ant predation on eggs in dispensers consisted of two compartments and involved only the species that had shown a preference for *E. kuehniella* eggs over seeds in the previous experiment. One ant colony was placed in a compartment, and insects were able to move through a transparent tube to a second compartment in which two *T*. *pretiosum* dispensers were available to them without other food resources (Figure 3). The experiment was again conducted at 23° C. Two devices were tested in parallel. In one case, the dispensers had 2 mm^2^ holes along the edges (usual dispensers, Figure 1A). In the second case, they had four holes smaller than 1 mm^2^ on both sides (modified dispensers, Figure 1B).

Egg density (number/mm^2^) within the dispensers was recorded before exposure to ants, 2 hr after the start of ant exposure, every hr up to 8 hr after initial exposure, and then again at 24, 48, and 72 hr after ant exposure. These measurements were performed in the same way as in the previous study.

### Laboratory and field tests to verify that *Trichogramma pretiosum* can easily emerge from modified dispensers and that the ants have little effect on them

The ability of *T. pretiosum* adults to emerge from the small holes of the modified dispensers was tested by placing five of these dispensers (Biotop), containing *E. kuehniella* eggs parasitized by *T. pretiosum*, in a clear plastic box located in a room maintained at 23° C. Ten days after the end of *T. pretiosum* emergence, the number of dead adults inside the box and the dispensers was counted.

These dispensers (Biotop) were used in January and February 2011 in an experimental field located near Dolores city, where four experimental releases against soybean pests were performed. Ten days after each release, 30 dispensers were collected from the field and transported to the laboratory. The number of dispensers attacked by ants and the number of dead *T. pretiosum* adults inside the dispensers were estimated.

### Statistical analysis

Data on ant behavior and density of host eggs in the dispensers were normally distributed, or in some cases almost normally distributed; therefore, parametric tests were used for the analyses. One-way repeated measures ANOVAs (time being the repeated variable) were carried out, with the factor “ant species” used for the analysis of ant movement towards the *E. kuehniella* eggs or towards the seeds as well as for the analysis of egg density in the first experiment. The factor “type of dispenser” was used for the analysis of egg density in the second experiment. Fisher PLSD tests were used post-hoc to separate ANOVA results by treatment.

**Figure 4.**
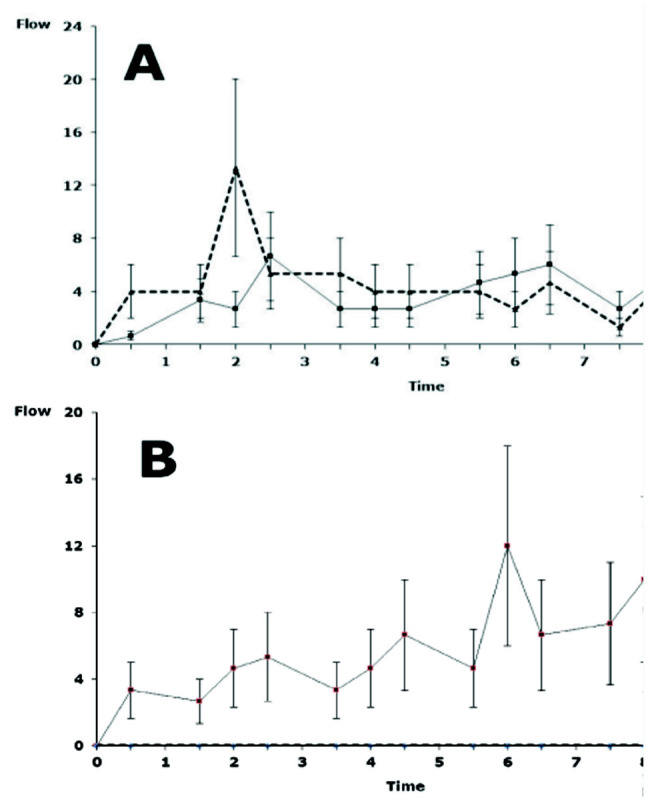
Flow of ants (number of individuals/min ± standarderror) over time (in hr) within transparent tubes leading to the*Trichogramma pretiosum* dispensers (triangles, broken line) or to the seeds (squares, continuous line). A: *Pheidole bergi*. B: *Solenopsis richteri*. High quality figures are available online.

## Results

### Identification of potential predators

The ants collected were identified as *Solenopsis* *richten* Forel (Hymenoptera: Formicidae) and *Pheidole bergi* Mayr. Among carnivorous ants, the black imported fire ant, *S. richteri*, is a very important species. It is widely distributed in Uruguay, southern Brazil, and the Argentine Pampas. *P. bergi* is also a carnivorous ant, and is widespread in the central parts of Argentina and in Uruguay (Kusnezov 1952; Kusnezov 1978a,b).

### Behavior of potential predators in the laboratory

During the first 2 hr of the experiment, *P. bergi* preferred the eggs of *E. kuehniella* over the seed mixture (Figure 4A). After the first 2 hr, the flow of ants moving towards the dispensers was essentially identical to that observed in the direction of the seeds. Figure 4B reveals that *S. richten* moved only in the direction of the seeds. During the 8.5 hr of observation, ant movement towards the dispensers was zero.

However, the ANOVA on movement towards eggs did not confirm the differences between the two ants (*F* = 4.00, df = 1, *p* = 0.183), despite differences over time (*F* = 4.00, df = 13, *p* = 0.001). The high variability of replicates could explain this lack of significance. The analysis of flow towards the seeds also indicates no difference between the ants (*F* = 0.48, df = 1, *p* = 0.558), again despite differences over time (*F* = 6.01, df = 13, *p* <0.0001).

The egg density within the dispensers visited by *P. bergi* was greatly reduced during the first hours of observation, as egg density decreased by more than half after 48 hr (Table 1). The egg density was unchanged over time when dispensers were exposed to *S. richten*. The ANOVA confirmed that the factor “species of ants” was significant (*F* = 57.54, df = 1 , *p* < 0.0001), with variations over time (*F* = 21.66, df = 8, *p* < 0.0001).

**Table 1.**
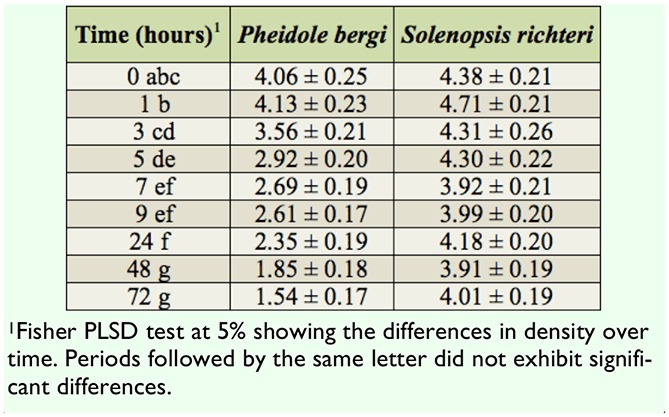
Host egg density per mm^2^ over time within the dispensers (N = 96) according to the ant species (*Pheidole bergi* or *Solenopsis richteri*) to which they were exposed.

### Laboratory tests of dispensers modified for the protection of *Trichogramma pretiosum*

This experiment was restricted to *P. bergi*, as it was the only ant species that expressed interest in dispensers and *E. kuehniella* eggs. The ANOVA showed that the type of dispenser (open on the edge or on sides) significantly influenced egg density (*F* = 98.57, df = 1, *p* < 0.0001), with variations over time (*F* = 19.38, df = 10, *p* < 0.0001). Egg density decreased greatly, especially in the early observation period, in the standard dispensers opened on the edge but not in the modified dispensers (Table 2).

Three hr after the start of the experiment, the egg density was reduced by 25% in the usual dispensers. This trend continued until the 5 hr after initial ant access and then slowed. At the end of the first day (24 hr), more than half of the *E. kuehniella* eggs had disappeared, and almost all the eggs were predated after three days.

### Laboratory and field tests verify that *Trichogramma pretiosum* easily emerged from modified dispensers and that the ants had little effect on *Trichogramma pretiosum*

In the laboratory, *T. pretiosum* adults easily emerged through the small holes of the modified dispensers. The number of insects found dead inside the dispensers was less than 5% of the total number of individuals counted inside the box (78 of a total of 1,750 *T*. *pretiosum* adults).

In the field, 30% of collected dispensers were preyed upon (36 of a total of 120 dispensers). Thus, ants were able to enlarge the small holes perforated in the dispensers. The number of *T*. *pretiosum* adults within the dispensers was very low.

**Table 2.**
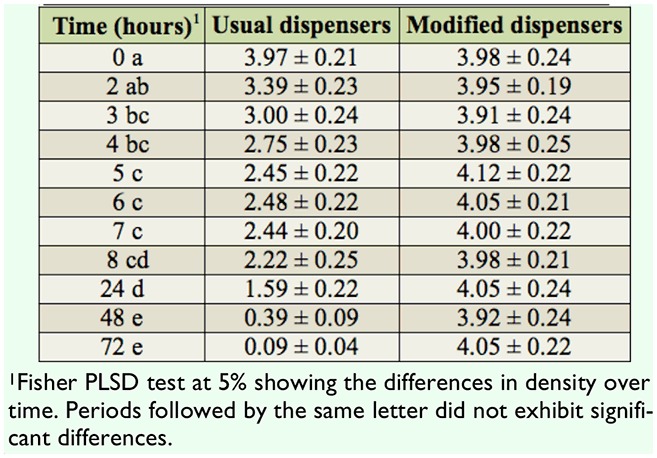
Host egg density per mm^2^ over time within the dispensers exposed to the ant *Pheidole bergi* (measurements were performed 32 times on each of the two dispensers of each type, and thus N = 64).

## Discussion

Only one of the two very common ant species present on soybeans in Uruguay that could be potentially responsible for predation of *T*. *pretiosum* in dispensers, *P. bergi*, exploited this food source. This result confirms the predatory voracity of that ant genus in ecosystems (Dejean et al. 2007), although a significant portion of its food is composed of seeds (Pirk et al. 2009). The literature indicates that species of *Solenopsis* can also cause problems in some biological control methods (Noldlund 1988; Hill and Hoy 2003; Tschinkel 2006), but the species *S. richteri* seemed harmless to *T. pretiosum* in the dispensers used in the present study. This ant did not penetrate into the dispensers and apparently did not detect the parasitized eggs. This genus, mainly carnivorous (Vogt et al. 2002), is an opportunist (Tschinkel 2006) that also feeds on nectar and seeds (Dress et al. 1991; Ready and Vinson 1995; Morrison et al. 1997).

The modified *T. pretiosum* packaging, which consisted of reduced diameter and altered position of the exit holes for the parasitoids, was effective, as the parasitized eggs were not predated. These modified dispensers are applicable in other geographical areas, as few predatory ants are smaller than those studied here. It is necessary to use *T. pretiosum* reared on eggs with a size similar to those of *E*. *kuehniella* or smaller. Other types of *T*. *pretiosum* protection could be considered, such as polyethylene bags, but because these are not biodegradable they present environmental problems (Marquier et al. 2009).

Field studies have shown that modified dispensers are still partially predated. An additional protection has therefore been developed by the company Biotop. It consists of gluing sticky tape on the dispensers at the level of the holes (Figure 1C). These dispensers were tested in the field in February–March 2012 under the same conditions as reported previously for dispensers without sticky tape. It was observed that the number of dispensers predated did not exceed 3%. This latter modification of dispensers has therefore been very effective. It was verified that the sticky tape did not trap *T. pretiosum* that fly when they flew through the holes of the dispensers. However, this modification may be only a temporary measure as the tape is not biodegradable.

## References

[bibr01] Al Rouechdi K, Voegelé J. (1981). Prédation des trichogrammes par les chrysopides.. *Agronomie*.

[bibr02] Castiñeiras A. (1981). Método para la cría y observación de dos especies de hormigas en condiciones de laboratorio.. *Ciencia y Técnica**en la Agricultura, Protección de Plantas*.

[bibr03] Dejean A, Moreau CS, Uzac P, Le Breton J, Kenne M. (2007). The predatory behavior of *Pheidole megacephala*.. *Comptes Rendus Biologies*.

[bibr04] Dress BM, Berger LA, Cavazos R, Vinson SB. (1991). Factors affecting sorghum and corn seed prédation by foraging red imported fire ants (Hymenoptera: Formicidae).. *Journal of Economic Entomology*.

[bibr05] Hill LS, Hoy AM. (2003). Interactions between the red imported fire ant *Solenopsis invicta* and the parasitoid *Lipolexis scutellaris* potentially affect classical biological control of the aphid *Toxoptera citricida*.. *Biological Control*.

[bibr06] Kusnezov N. (1952). El género *Pheidole* en la Argentina (Hymenoptera, Formicidae).. *Acta**zoológica lilloana*.

[bibr07] Kusnezov N. (1978a). *Hormigas argentinas:**clave para su identificación. Parte 1*..

[bibr08] Kusnezov N. (1978b). *Hormigas argentinas:**clave para su identificación. Parte 2*..

[bibr09] Marquier M, Roux E, Frandon J, Goebel R, Tabone E. (2009). Les fourmis: prise en compte de leur action pour lutter contre le foreur de la canne à sucre, *Chilo sacchariphagus* Bojer.. *Colloque International sur la Gestion des**Risques Phytosanitaires, Marrakech, Maroc*.

[bibr10] Ministerio de Ganadería, Agricultura y Pesca [MGAP] (2011). *Historical series*..

[bibr11] Mgocheki N, Addison P. (2009). Interference of ants (Hymenoptera: Formicidae) with biological control of the vine mealybug *Planococcus ficus* (Signoret) (Hemiptera: Pseudococcidae).. *Biological Control*.

[bibr12] Morrison JE, Williams DF, Oi DH, Potter KN. (1997). Damage to dry crop seed by red imported fire ant (Hymenoptera: Formicidae).. *Journal of Economic Entomology*.

[bibr13] Noldlund DA., Vinson SB, Teer J (1988). The imported fire ant and naturally occurring and released beneficial insects.. *Proceedings of the Governor's Conference on**the imported fire ant: assessment and recommendations*.

[bibr14] Pirk GI, di Pasquo F, Lopez de Casenave J. (2009). Diet of two sympatric *Pheidole* spp. ants in the central Monte desert: implications for seed-granivore interactions.. *Insectes**Sociaux*.

[bibr15] Ready CC, Vinson SB. (1995). Seed selection by the red imported fire ant (Hymenoptera: Formicidae) in the laboratory.. *Environmental**Entomology*.

[bibr16] Smith SM., Wajnberg E, Hassan SA (1994). Methods and timing of releases of *Trichogramma* to control Lepidopterous pests..

[bibr17] Tschinkel WR. (2006). *The fire ants*..

[bibr18] van Lenteren JC., Gurr B, Wratten S (2000). Measure of success in biological control of arthropods by augmentation of natural enemies.. *Measures of Success in Biological Control*.

[bibr19] van Lenteren JC, Bueno VHP. (2003). Augmentative biological control of arthropods in Latin America.. *BioControl*.

[bibr20] Vogt JT, Grantham RA, Corbett E, Rice SA, Wright RE. (2002). Dietary habits of *Solenopsis*
*invicta* (Hymenoptera: Formicidae) in four Oklahoma habitats.. *Environmental**Entomology*.

